# Recent Update on the Role of Chinese Material Medica and Formulations in Diabetic Retinopathy

**DOI:** 10.3390/molecules22010076

**Published:** 2017-01-04

**Authors:** Sandeep Vasant More, In-Su Kim, Dong-Kug Choi

**Affiliations:** Department of Biotechnology, Konkuk University, Chungju 380-701, Korea; sandeepbcp@gmail.com (S.V.M.); kis5497@kku.ac.kr (I.-S.K.)

**Keywords:** diabetes, herbal, medicine, retinopathy, traditional

## Abstract

Diabetes mellitus is one of the most frequent endocrine disorders, affecting populations worldwide. Diabetic retinopathy (DR) is the most frequent microvascular complication of diabetes in patients aged 20 and over. Major complications of DR include intraocular neovascularization, inter-retinal edema, hemorrhage, exudates and microaneurysms. Therefore, timely medical attention and prevention are required. At present, laser-assisted therapy and other operational procedures are the most common treatment for DR. However, these treatments can cause retinal damage and scarring. Also, use of the majority of traditional medicines is not supported by clinical evidence. However, due to accumulating scientific evidence, traditional natural medications may assist in delaying or preventing the progression of DR. This review focuses on evidence for the role of traditional natural medicines and their mechanisms of action and pharmacological test results in relation to the progression of DR.

## 1. Introduction

Diabetes is now a global concern, with recent surveys forecasting that by 2030, the number of patients with diabetes will increase to 500 million [1]. Morbidity and premature deaths are caused by macrovascular (cardiovascular, cerebrovascular, and peripheral vascular disease) and microvascular (neuropathy, nephropathy, and retinopathy) diabetic complications. Diabetic retinopathy (DR) is one of the leading reasons for visual impairment and blindness in the working-age population [2]. DR can be defined as a pathology of the capillaries, arterioles and venules in the retina and the subsequent effects of leakage from, or occlusion of, the small vessels [3]. Retina is a specialized tissue responsible for vision, which processes visible light into the neuronal signals that can be perceived by the brain. The distinctive vascular system of retina facilitates oxygen and nutrients to the outer and inner retina, whose integrity is crucial for sensing light [4]. Thus, the vascular retinal structure obtains blood-retinal barrier (BRB) characteristics, which are essential for the integrity of vascular retinal structure and the maintenance of the retinal microenvironment. Malfunctioning of vascular cells in inner BRB during DR causes breakdown of the BRB, which leads to vascular leakage and subsequent macular edema, ultimately causing serious impairment of vision [4].

According to the World Health Organization, DR accounts for 4.8% of the 37 million cases of blindness worldwide [5]. Epidemiological clinical studies have reported risk factors for DR progression [6,7]. High blood pressure, hyperglycemia, and duration of diabetes are the major risk factors for DR. In the initial 3–5 years, retinopathy is rare in type I diabetic patients. However over the age 20 years, 90%–95% of patients exhibit some degree of retinopathy [8,9]. In contrast, almost 60%–70% of type II diabetic patients have some retinopathy after 20 years [10]. Other factors associated with DR include gender, ethnicity, age at onset of diabetes, cataract extraction, puberty, and pregnancy [2]. Genetics may also be implicated in DR, since some patients with a long duration of diabetes fail to develop DR while, despite tight blood glucose control, some patients still rapidly develop DR [11,12]. The majority of diabetic patients do not experience any precautionary symptoms during the early stage of DR. The anatomical alterations that take place during DR have been well distinguished and include formation of acellular capillaries, the loss of pericyte and endothelial cells, formation of microaneurysms and retinal neovascularization and early thickening of the basement membrane [13]. Additionally, hard exudates, cotton wool spots, venous abnormalities, formation of microaneurysms, dot and blot retinal hemorrhages, and the growth of new blood vessels are some of the clinical signs of retinopathy [14]. Nonetheless, symptoms that may imply urgent discussion with an eye care professional include the presence of floaters and flashes and blurred or sudden loss of vision. Timely identification of DR can avoid severe loss of vision and blindness. Clinical identification of DR includes examination of fundus, retinal photography and visual acuity test. Diabetes involves a series of pathological factors such as hormones, hyperglycemia, hypertension, inflammation and growth factors that insinuate harmful biochemical pathways such as oxidative stress, apoptosis and inflammation accountable for the development of the disease. The pathways and their mediators ultimately damage both neuronal and vascular cells in the retina, leading to neurovascular damage in DR [15]. Non-proliferative DR (NPDR) and proliferative DR are the two main stages of DR. Diabetic macular edema (DMO), caused by leakage and build-up of fluid and proteins within two disc diameters of the macular region, in combination with proliferative DR is a major cause of severe visual impairment in patients with diabetes [16]. Further, there are three types of DMO—edematous, ischemic and exudative [2]. Strict control over blood pressure, blood glucose, and probably lipids are vital management strategies to decrease the onset and progression of DR [2]. Treatment for DR involves use of ACE inhibitors [17] and surgical procedures such as laser photocoagulation and possibly vitrectomy [18,19].

## 2. Status of Traditional Herbal Medicine in Diabetic Retinopathy

In modern times, there has been mounting interest in unconventional therapies and the therapeutic use of traditional herbal medicines [20]. More than 400 traditional medicinal plants and more than 800 active herbal compounds are documented to have therapeutic potential for diabetes [21,22,23]. For centuries, herbal and traditional medicine (TM) has been used worldwide for treating diabetes and its complications. Additionally, TM has potential as an alternative therapy to delay or prevent the progression of DR [24]. Based on these reports, research has been undertaken to explore the mechanism of action of TM and its active compounds and also how to transform these active principles into new treatments for improved management of diabetes and its complications including DR [25]. The broadness of “TM” and the extensive range of practices it includes make it difficult to describe, especially in a global context. TM is a significant and often underrated part of health services. In several countries, TM is often referred to as complementary medicine. TM has been used from ancient times for the prevention and treatment of chronic disease and also in health maintenance [26]. Use of TM is steadily increasing in countries where its use was not recognized [26]. Knowledge of TM may be passed on verbally from generation to generation, in some context with families concentrating on specific treatments, or it might be taught officially in renowned universities. Occasionally practice of TM is limited to some specific geographical areas; however, it may also be observed in diverse regions of the world. However, in the majority of cases, a medical system is only termed “traditional” when it is exercised inside the country of origin.

**Figure 1:**
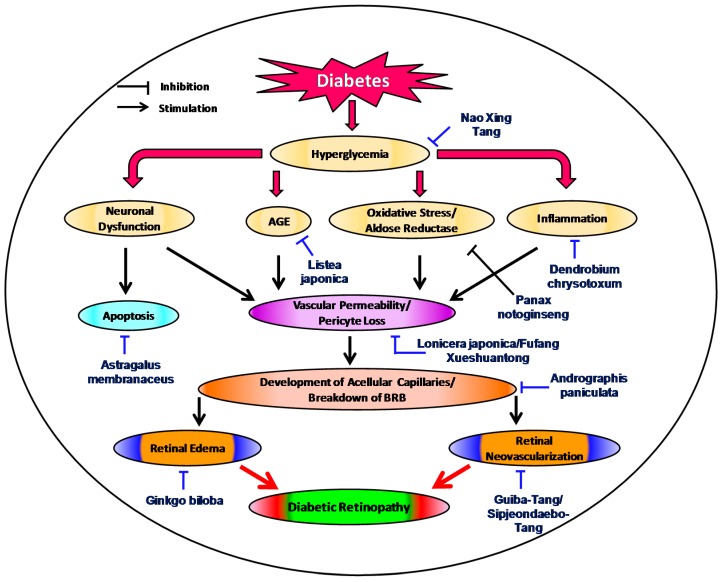
Proposed schematic presentation of the signaling pathways in the pathophysiologic development of diabetic retinopathy [32,33,34,35,36,37,38]. The diagram illustrates traditional medicinal plants or formulations targeting each of these respective pathways that might attenuate the progression of diabetic retinopathy.

Various countries have their own native or traditional forms of curing which are decisively rooted in their history and culture. Some forms of TM such as Ayurveda, Unani and Traditional Chinese Medicine are popular nationally and also well recognized and practiced worldwide [26]. Several factors led to the dissemination and rising demand of TM throughout the world, mainly in the past 20 years. TM is more accessible conventional medicine in a few areas. Nonetheless, the most frequent reasons for using TM are that it is more reasonably priced; more closely related to the patient’s ideas, and is less paternalistic than allopathic medicine. Irrespective of the reasons why an individual uses it, TM provides a crucial health care service to people both with and without financial means or geographic access to allopathic medicine [27]. Considering the history and applicability of TM, it should be subjected to a rigorous drug discovery process to yield a reliable drug candidates. At present, drug discovery is no longer a process limited to the accessibility of new technology [28]. During the past decade a considerable number of accepted new drug applications have been derived from the biotechnology industry and analysts anticipate a similar trend in future [29]. Correspondingly, natural products have contributed almost half of all small molecules drugs developed in this decade. It would be highly desirable and helpful for drug discovery, if the drug discovery process were shifted to combinations of existing agents. Therefore, drug discovery of natural products based on TM and ethnopharmacology might also be useful strategic options [30]. One of the validated approaches to drug discovery and development of TM are bioactivity-guided isolation and structural elucidation, by which we can discover a new molecule for a precise disease target. Numerous Western drugs, such as paclitaxel, were discovered in this way, whereas others drugs, such as metformin, were developed by further structural modification. Another approach is to combine different herbs or ingredients with proper quality control and fine tune the dose regimen and reproducibility to obtain a desired outcome for a particular disease in the general patient population [31]. Herein, we discuss the scientific evidence supporting the role of traditional natural medicines (Figure 1) and their mechanisms of action, and the results of pharmacological testing in relation to the progression of DR.

## 3. Recent Evidences of Traditional Medicinal Plants in Diabetic Retinopathy

### 3.1. Litsea japonica

*Litsea japonica* (*L. japonica*) is an important native Korean plant species [39]. This herb has been consumed as a vegetable food in Korea; however, its pharmacological activities are unclear. The chemical constituents of this plant comprise numerous lactones, alkaloids, essential oils, fatty acids, and terpenoids [39,40]. Treatment with an extract of *L. japonica* inhibited diabetes-induced breakdown of BRB and lowered expression of retinal vascular endothelial growth factor (VEGF) in *db/db* mice. Extract of *L. japonica* also suppressed the degradation of occludin, which is an important tight junctional protein in BRB. These results suggest the potential therapeutic usefulness of *L. japonica* for retinal vascular permeability diseases [41]. Later, Kim and colleagues explored the ameliorative effect of an ethanolic extract of *L. japonica* on diabetes‑induced neuronal apoptosis of the retina in *db/db* mice. Treatment with an ethanolic extract of *L. japonica* (100 or 250 mg/kg body weight) once per day orally for 12 weeks in *db/db* mice resulted in a trivial decrease in blood glucose levels without any significant effect on those of HbA1c. Immunoreactivity against advanced glycation end product (AGE) was evident only in the large and small retinal vessels of the normal mice, while AGE-positive signals were situated in the inner neural retina and retinal vessels in the vehicle-treated *db/db* mice. This suggests that retinal tissues exhibit significant accumulation of serum AGEs. However, treatment with ethanolic extract of *L. japonica* reduced the amount of AGE deposited in these regions. The suppressive effect of ethanolic extract of *L. japonica* on the expression of RAGE was also examined. The immunoreactivity against RAGE was higher in vehicle-treated *db*/*db* mice compared with normal mice. However, treatment with ethanolic extract of *L. japonica* lowered RAGE in the neural retinas of the *db/db* mice. Treatment with an ethanolic extract of *L. japonica* also significantly decreased the number of TUNEL-positive cells in the ganglion cell layer and the inner nuclear layer. In addition, the ethanolic extract of *L. japonica* inhibited the stimulation of nuclear factor-kappa B (NF-κB). These results suggested that the ethanolic extract of *L. japonica* may be advantageous for the treatment of diabetes-induced retinal neurodegeneration; moreover, its neuroprotective effect might be attributable in part its effect on AGEs [32].

### 3.2. Ginkgo biloba

*Ginkgo biloba* is a well-known Chinese TM used normally for respiratory disorders, and to enhance memory loss-related abnormalities of blood circulation in Iran [42]. Quercetin is also the chief bioactive ingredient of *Ginkgo biloba* and St. John’s Wort [34]. Quercetin (3,5,7-trihydroxy-2-(3,4-dihydroxyphenyl)-4*H*-chromen-4-one) is one of the most powerful, commonly studied dietary flavonoid, which is frequently encountered in seeds, barks, flowers, tea, brassica vegetables, onion, apple, berries, many nuts, and leaves [43]. Quercetin is documented to have many biological functions including anti-diabetic, anti-carcinogenic, cataract prevention, anti-inflammatory, anti-ulcer effects, anti-allergic activity, and anti-viral activities [44]. Furthermore, it blocks aggregation of platelets, capillary permeability and lipid peroxidation too. Due to its efficacious anti-oxidant profile, it inhibits xanthine oxidase, directly scavenges free radicals, changes antioxidant defence and suppresses lipid peroxidation [45]. Prevention of cataract, inhibition of choroidal and retinal angiogenesis [46] and prevention of oxidative damage in retinal pigment epithelium cell cells [47] are a few of the bioactivities of quercetin. Studies performed on green tea and *Moringa oleifera* (both of which contain quercetin) established the background that quercetin might play a protective role in DR [48,49]. Treatment with quercetin (25 and 50 mg/kg body weight) reversed the reduced retinal GSH levels in streptozotocin (STZ)-induced diabetic rats. Treatment with quercetin considerably lowered the levels of cytokines in the diabetic retina of STZ-intoxicated rats. Light microscopy indicated a significant increase in ganglion cell death and reduced retinal thickness in the diabetic compared to the normal retina. Conversely, treatment with quercetin abolished this effect. Quercetin demonstrated attenuated the expression NF-κB and caspase-3. Further, administration of quercetin ameliorated diabetes-induced increases in glial fibrillary acidic protein and aquaporin 4 expression and thereby reduced the upregulated glial fibrillary acidic protein P expression and edema in their endfeet and around the perivascular space in the nerve fiber layer of the diabetic retina [34]. Therefore, quercetin might be effective against DR, which affects both vascular and neural components of the diabetic retina.

### 3.3. Pueraria lobata

Puerarin is a well-known isoflavone glycoside obtained from the root of *Pueraria lobata* that has been used in Korean TM to treat a variety of diseases [50]. Puerarin is also a major active principle extracted from the traditional Chinese medicine known as Ge-gen [51]. Due to its protective effects on pancreatic islets and stimulation of insulin sensitivity, puerarin has been extensively investigated as an antihyperglycemic agent [52,53]. Furthermore, puerarin successfully restrains AGE formation [54] and is generally accepted to play a benevolent role in DR, but its mechanism remains poorly understood. Lately, studies performed by Kim found puerarin (10 µM) to suppress apoptosis in pericytes induced by AGE-bovine serum albumin. Puerarin (10 µM) clearly blocks the generation of ROS induced by AGE-bovine serum albumin. Puerarin markedly inhibits generation of reactive oxygen species (ROS) via the inhibition of nicotinamide adenine dinucleotide phosphate oxidase. Further mechanistic studies found that puerarin blocks AGE-bovine serum albumin-induced phosphorylation of Rac1 and p47phox and thereby attenuates ROS production via nicotinamide adenine dinucleotide phosphate [55]. Administration of puerarin significantly decreases the activation of NF-κB. Besides, puerarin also attenuates the in vivo apoptosis of retinal pericytes induced by intravitreal injection of AGE-rat serum albumin. These data suggest that puerarin might exert its beneficial effects on AGE-induced pericyte apoptosis by modulating the nicotinamide adenine dinucleotide phosphate oxidase-related ROS pathways and by inhibiting NF-κB activation to avoid dysfunction in the retinal microvasculature [55]. A protective effect of puerarin was also seen in retinal pigment epithelial cells. In this report by Wang and colleagues, puerarin was found to reduce the apoptosis of retinal pigment epithelial cells in STZ-induced diabetic SD rats by suppressing the peroxynitrite level and expression of inducible nitric oxide synthase [56]. A similar beneficial effect of puerarin was observed in STZ-induced DR rats. In this study, puerarin significantly decreased morphological changes of the inner nuclear layer and outer nuclear layer of the retina. Additionally, puerarin also regulates the expression of hypoxia-inducible factor-1α and VEGF induced by STZ.

### 3.4. Lonicera japonica

*Lonicera japonica* (*L. japonica*) is a native Chinese TM also known as *Jin Yin Hua*. *L. japonica* has been documented to possess various biological activities including antiviral, anti-inflammatory and antibacterial, antipyretic and blood fat-reducing activities [57]. Chlorogenic acid (CGA) is one of the major constituents of *L. japonica* [58]. CGA, formed by esterification of caffeic and quinic acids, belongs to the class of polyphenols commonly found in beans, potatoes, apples, and coffee. Various studies have documented the anticarcinogenic, anti-inflammatory, antioxidant and antibacterial activities of CGA [59,60,61]. A number of mechanisms have been proposed for the beneficial effects of CGA on glucose metabolism. CGA might impede glucose absorption in the small intestine [61], and decrease hepatic glucose output [62,63]. Moreover, due to its metal chelator [64] and antioxidant effects, CGA might prevent the development of glucose intolerance and insulin resistance. Nevertheless, the role of CGA in DR has not yet been determined. A recent study by Shin and associates evaluated the effect of CGA in the rat model of DR [56]. In this study, CGA exerted a dose-dependent beneficial effect in STZ-induced DR in diabetic rats. CGA efficiently obstructs the increase in VEGF and decrease in tight junctional proteins, including occludin, without any effects on the levels of claudin-5. CGA also reduced the increased VEGF levels in the diabetic retina. Based on these results, CGA can be recommended as a supplementary treatment for DR [33].

### 3.5. Andrographis paniculata

Andrographolide is a naturally occurring diterpenoid lactone and the chief compound isolated from the traditional medicinal herb *Andrographis paniculata* (*A. paniculata*) [65]. *A. paniculata* is famous for its detoxifying effect and reducing temperature. It has also been in common use for centuries in Asian countries for the treatment of upper respiratory tract infections and sore throat [65]. Andro is reported to have potent anti-inflammatory effects in various experimental models of pulmonary fibrosis, inflammatory bowel disease, asthma and cigarette smoke-induced lung injury [55,66,67,68]. In a very recent report Yu and colleagues explored the effect of andrographolide on STZ-induced DR in C57BL/6 mice. Data obtained from retinal immunofluorescence staining with cluster of differentiation-31 demonstrated that andrographolide decreased the excessive growth of retinal vessels in STZ-induced proliferative DR mice. Studies exploring permeation via Evans blue found that andrographolide mitigated BRB breakdown in STZ-induced NPDR mice. Andrographolide diminished the elevated levels of VEGF in serum and the vitreous cavity, and also subdued the augmented mRNA expression of retinal VEGF and its receptors in STZ-induced proliferative DR mice. In contrast, in STZ-induced NPDR mice, andrographolide suppressed the nuclear translocation of early growth response-1 and, p65 NF-κB and further decreased the phosphorylation of p65-NF-κB, inhibitor of kappa B, and inhibitor of kappa B kinase. STZ-induced serum and retinal mRNA expression of tumor necrosis factor-α (TNF-α), interleukin-6 (IL-6), interleukin-1β (IL-1β), serpine1, and tissue factor were also found to be diminished by andrographolide. In conclusion, these results suggest a noticeable effect of andrographolide on angiogenesis and retinal inflammation during the early phases of DR, in which signaling related to VEGF, NF-κB, and early growth response-1 plays a vital role. This study provides evidence for the clinical utility of andrographolide for the treatment of DR [35].

### 3.6. Astragalus membranaceus

Astragaloside IV (AS-IV) is the pure primary and major saponin isolated from the root of *Astragalus membranaceus*. AS-IV is an effective compound with distinct pharmacological effects, including anti-inflammatory [69] and regulation of immunological function [70], antioxidation [71], and protection from live injury [72]. Interestingly, AS-IV is reported to inhibit glycogen phosphorylase, glucose-6-phosphatase and formation of AGE and thereby exerts a hypoglycemic effect in diabetic mice [73,74]. Moreover, AS-IV has also displayed protective effects on peripheral diabetic neuropathy in rats [75]. Nonetheless, the protective effect of AS-IV on DR has not been examined to date. Based on the observations described above, Ding et al. explored the inhibitory effects and signaling mechanism of AS-IV on DR in type 2 diabetic *db/db* mice. Treatment with of AS-IV considerably enhanced the pattern electroretinogram amplitude and decreased the apoptosis of retinal ganglionic cells in *db/db* mice. Additionally, administration of AS-IV caused downregulation of AR activity, and phosphorylation of extracellular signal-regulated kinase1/2 and NF-κB in *db/db* mice [36]. In conclusion, these results established the neuroprotective and anti-inflammatory ability of AS-IV in *db/db* mice with DR. Although further clinical trials are required to demonstrate the clinical efficacy of AS-IV, it can be hypothesized that AS-IV is an inhibitor of AR and might be useful for the treatment of DR.

### 3.7. Salvia miltiorrhiza

*Salvia miltiorrhiza* (Danshen in Chinese) is a frequently used traditional Chinese herbal medicine. Compound danshen dripping pill (CDDP) is a danshen-containing Chinese herbal medicine product that has been used to treat cardiovascular diseases [76]. This pill is a mixture of three traditional Chinese medicines namely, danshen, notoginseng and borneol, that have been used for centuries for the treatment of various ailments. CDDP has been reported to aid blood circulation and assuage pain [76]. With respect to Chinese TM, the pathogenesis of DR is due mainly to blood stasis, which damages collateral vessels in the eye [76]. In line with this, various experimental studies and clinical DR trials in DR patients have established that CDDP can ameliorate the symptoms of DR [77]. Lian and associated recently reported a large-scale clinical trial involving 223 NPDR patients that planned to evaluate the efficiency and safety of CDDP in patients with NPDR [78]. The duration of the trial was 24 weeks and patients were divided into four groups namely, placebo, low-dose (270 mg), mid-dose (540 mg) and high-dose (810 mg herbal medicine) groups. Data obtained from fluorescence fundus angiography at 24 weeks indicated that the percentage of “excellent” and “effective” subjects in the mid- and high-dose CDDP groups was significantly higher (77% and 74%) than that in the placebo group (28%). Similarly, fundoscopic examination revealed that the percentage of “excellent” and “effective” subjects in the mid- and high-dose CDDP groups was significantly higher (42% and 59%) than that in the placebo group (11%). Episodes of adverse events with clinical significance were not seen [78]. This clinical trial thus confirms the efficacy and safety of a danshen-containing Chinese herbal medicine in patients with DR.

### 3.8. Dendrobium chrysotoxum

*Dendrobium chrysotoxum* (DC) is a well-recognized herbal Chinese TM found in the mountain ranges of western and southern China [79]. DC is reported to possess various bioactivities, including antioxidant, immunomodulatory, anticancer, and anti-senescence activities [80]. Moreover, polysaccharides isolated from DC have antihyperglycemic and antioxidant effects [81]. Yu and coworkers reported positive effects of ethanolic extract of DC on retinal angiogenesis during the early phases of DR via blocking the expression of VEGF/VEGF receptor 2 and other pro-angiogenic factors, such as basic fibroblast growth factor, matrix metaloproteinase-2/9, platelet-derived growth factor-A/B, and insulin growth factor-1. Moreover, DC mitigates NF-κB-induced retinal inflammation in DR [38]. In a similar study by Yu and coworkers, DC attenuated STZ-mediated destruction of the BRB. STZ-induced mRNA expression of tight junction proteins including occludin and claudin-1, intercellular adhesion molecule-1 (ICAM-1), TNF-α, IL-6, and IL-1β was also decreased by DC in diabetic rats. Further retinal immunofluorescence staining and western blot analysis revealed that DC normalizes the expression of occludin and claudin-1 proteins in diabetic rats. In addition, DC diminishes the phosphorylation level of p65-NFκB, inhibitor of kappa B, and inhibitor of kappa B kinase in diabetic rats. DC also abated the STZ-induced serum levels of TNF-α, interferon-γ, IL-3, IL-6, IL-8, IL-10, IL-12 and IL-1β in diabetic rats [82]. The ability of DC to improve eyesight suggests its potential for use in patients with DR.

### 3.9. Vaccinium myrtillus

*Vaccinium myrtillus* (*V. myrtillus* (bilberries), *Vaccinium cyanococcus* (blueberries) and blackcurrants are berry fruits that contain plentiful anthocyanins, making them major dietary sources of anthocyanin [83,84]. Substantial consideration has focused on the health benefits of bilberry, which include anti-neurodegenerative, anti-inflammatory, antioxidant, and anticancer activities [85]. Extract of *vaccinium myrtillus* (VME) comprises a mixture of 15 anthocyanins [86]. Animal studies have suggested that anthocyanins from *V. myrtillus* improve blood flow, vascular tone, and vasoprotection [87,88]. It has been reported that VME blocks VEGF-mediated angiogenesis of retinal vasculature and proliferation of human umbilical vein endothelial cells in oxygen-induced retinopathic (OIR) mice by suppressing the phosphorylation of protein kinase B and extracellular signal regulated kinase 1/2 (ERK 1/2) [89]. Kim et al. determined whether *vaccinium. myrtillus* (100 mg/kg) could prevent STZ-induced dysfunction of the retinal vasculature in rats. In situ hybridization of retinal cells revealed that the increased retinal mRNA VEGF levels were considerably lowered by VME. Fluorescein-dextran angiographic studies revealed that the leakage of fluorescein was noticeably decreased in diabetic rats treated with VME. VME treatment also reinitiated the expression of endothelial cell-to-cell junction proteins, particularly zonula occludens-1, occludin and claudin-5, in diabetic rats [90]. Hence, these results suggest that VME blocks diabetes-mediated BRB breakage in diabetic rats by preventing VEGF expression. Therefore, VME may contribute to prevention of diabetes-induced BRB breakdown.

### 3.10. Zingiber zerumbet

*Zingiber zerumbet* (*Z. zerumbet*), also known as wild ginger, is a profitable spice known as Hong qiujiang in Taiwan. *Z. zerumbet* is usually grown in village gardens in the tropics for its medicinal properties [91]. Regardless of its regular uses as an appetizer and food flavoring agent, rhizomes of *Z. zerumbet* (ZZR) have been used traditionally for medicinal purposes including colds, ulcers, sores, headaches, swelling, loss of appetite, nausea and even menstrual discomfort in Chinese, Indian, and Arabic cultures since ancient times [92]. Quercetin, kaempferol, curcumin, 2,6,9-humulatrien-8-one, humulene and camphene are constituents of ZZR [93]. Since ZZR promotes glucose homeostasis, it can be used as a supplemental therapy for diabetes and its associated complications, including DR. Conversely, attenuation of DR by ZZR has not been examined. Hong and associates recently reported that treatment of STZ-diabetic rats with ethanol extract of ZZR reinstated the retinal vascular permeability to a significant extent as compared to the normal diabetic group. Treatment with an ethanol extract of ZZR increased the thickness of the ganglion cell layer, inner nuclear layer, outer nuclear layer and nerve fiber layer in STZ-diabetic rats. The ethanol extract of ZZR reversed the decreased retinal expression of VEGF and stimulated the expression of renal pigment epithelium-derived factor in diabetic rats. Treatment with the ethanol extract of ZZR also reduced the mRNA expression of intercellular adhesion molecule-1, vascular cell adhesion molecule-1 TNF-α, IL-1, IL-6 and monocyte chemotactic proteins-1. Furthermore, ethanol extract of ZZR could inhibit phosphorylation of extracellular signal-regulated kinase-1/2 and nuclear localization of p65-NF-κB [94]. Therefore, re-establishing the balance between inhibitors and stimulators of angiogenesis might be associated with the beneficial effect of the ethanol extract of ZZR on DR.

### 3.11. Trigonella foenum-graecum

*Trigonella foenum-graceum*, also known as fenugreek, is a well-recognized herbal plant that is used as a vegetable and spice on the Indian subcontinent. Trigonelline, 4-hydroxyisoleucine and saponins are the chief constituents of fenugreek. Traditionally, fenugreek exhibits antidiabetic [95,96], anti-hyperlipidemic [97], anti-inflammatory [98], antioxidant [99], and neuroprotective [100] properties. Recently, Gupta and associates examined the effects of fenugreek in STZ-induced diabetic complications in the rat retina. Injection of STZ to Wistar rats considerably elevated the expressions of retinal inflammatory (IL-1β and TNF-α) and angiogenic (VEGF and PKC-β) molecular biomarkers in diabetic retinae in comparison to normal retinae. Treatment with fenugreek (100 and 200 mg/kg body weight) for 24 weeks inhibited the expression of inflammatory and angiogenic molecular biomarkers. Moreover, fenugreek reinstated normal superoxide dismutase and catalase levels in the diabetic retinae. Fluorescein angiograms and photographs of the fundus of diabetic retinae indicated retinal vascular leakage. However, this effect was reversed in fenugreek-treated retinae. Fenugreek also decreased the thickened basement membrane in STZ-induced diabetic retinae. Hence, fenugreek may prevent retina degeneration in diabetic patients [34].

### 3.12. Guibi-Tang

Guibi-tang (GBT) is an herbal TM in used in Asian countries. Guibi-tang is a combination of 12 herbs that are used to treat poor memory, anorexia, amnesia, fatigue, palpitation, neurosis anemia, and insomnia [101]. GBT has also been patented for use in wet macular degeneration [102]. Fresh data have suggested that GBT has various bioactivities, namely antioxidant [103], anti-stress [104], protection of the gastric mucosa [105] and immune regulation [106]. One of the major ingredient in GBT is decursin, which inhibited retinal neovascularization in a mouse model of retinopathy in prematurity [37]. Irrespective of the effects of GBT, information on its underlying mechanisms during retinal neovascularization is limited [107]. Lee and associates examined the effect of GBT on pathogenic retinal neovascularization in a mouse model of oxygen-induced retinopathy. Intraperitoneal administration of GBT (50 or 100 mg/kg/day) in OIR C57BL/6 mice significantly reduced pathogenic angiogenesis of the retina, and the protein level of plasminogen activator inhibitor-I. Real-time PCR demonstrated that GBT lowered mRNA levels of VEGF, fibroblast growth factor-2, and plasminogen activator inhibitor-1 in OIR mice [107]. Therefore, GBT exerts anti-angiogenic effects through partial inhibition of fibroblast growth factor-2, plasminogen activator inhibitor-1 and VEGF. Further research is necessary to assess the clinical potential of GBT.

### 3.13. Samul-Tang

Samul-tang (SMT) is a well-known decoction in Chinese TM [108,109]. This decoction comprises four medicinal herbs namely, *Angelica gigas*, *Cnidium officinale*, *Paeonia lactiflora* and *Rehmannia glutinosa* [110]. SMT has long been extensively utilized in Eastern Asia to alleviate pain, blood deficiencies, gynecological ailments and chronic inflammation, and increase blood circulation [111,112]. In contrast, SMT exerts biological effects including, anti-pruritic, anti-inflammatory, and anticancer [112], anti-angiogenic [113], and anti-diabetic [114] properties. However, the effect of SMT on retinal neovascularization or pathogenic retinal angiogenesis has not been reported [110]. Lee and colleagues reported that SMT reduced the area of the central retina and reduced retinal neovascularization in OIR mice. SMT (10 or 50 mg/kg) once per day for five days decreased the protein level of stromal cell-derived factor-1. Administration of SMT significantly lowered the increased mRNA levels of stromal cell-derived factor-1, C-X-C chemokine receptor type 4, hypoxia-inducible factor-1α and VEGF in the retinas of OIR mice [110]. These data suggest that SMT modulates hypoxia-inducible factor-1α and acts via an anti- stromal cell-derived factor-1/C-X-C chemokine receptor type 4 and anti-VEGF mechanism to prevent pathogenic angiogenesis of retinal cells. These findings suggest SMT to be a valuable herbal TM for treating ischemic retinopathy.

### 3.14. Fufang Xueshuantong

Based on the meridian theory of TCM, Fufang Xueshuantong (FXT) capsule was originally formulated twenty years ago and also has been accepted in 2003 by the State Food and Drug Administration of China for treatment of stable angina pectoris and retinal vein occlusion [115,116]. Fufang Xueshuantong (FXT), also known as compound Xueshuantong, is a Chinese herbal formula that consists of four traditional plants, namely *Astragalus membranaceus, Scrophularia ningpoensis, P. notoginseng* and *Salvia miltiorrhiza* [117]. FXT is used clinically to treat DR [118]. Moreover, FXT attenuates the progress of microvessel lesions in the retina by diminishing pericyte loss and decreasing acellular capillaries. This effect was in agreement with the upregulation of occludin and downregulation of aldose reductase hyperactivity and expression of VEGF and ICAM-1. Thus, FXT exhibited protective effects against STZ-induced retinal lesions in rats [119]. Constituent combination of FXT (cFXT) is composed of saponins of *P. notoginseng*, harpagoside, cryptotanshinone, tanshinone-I, and gastragaloside-A. Although cFXT had no effects on the STZ-induced augmented blood glucose levels and body weight in rats, it inhibited the augmented erythrocyte aggregation, plasma viscosity, and pericyte and acellular vessel loss. This was accompanied by reversal of the increased expression of VEGF, ICAM-1, and endothelin-1, excessive activation of aldose reductase, and hypoexpression of pigment epithelium-derived factor and occludin in the retinas of STZ-treated rats [120]. Hence, the main constituents of Fufang Xueshuantong Capsule, namely saponins of *P. notoginseng*, harpagoside, cryptotanshinone, tanshinone-I, and astragaloside-A are responsible for its protective effects in STZ-induced retinal lesions in rats. These constituents will facilitate development of new drug for DR, and can be used for quality control of Fufang Xueshuantong Capsules [120].

### 3.15. Ligusticum chuanxiong Hort

*Ligusticum chuanxiong Hort (chuanxiong*) is a well-known Chinese TM that has been extensively used for neurovascular and cardiovascular diseases in China. Tetramethylpyrazine (TMP) is one of the major active alkaloid constituents of *Ligusticum chuanxiong Hort* [121] used in Asian countries for hundreds of years to treat heart, kidney, and brain diseases [122]. Previous studies have demonstrated that TMP possesses different pharmacological activities, including free radical scavenging [123], anti-inflammation [124], and calcium antagonism [125]. Systematic administration of TMP has been documented to protect against spinal cord injury and ischemia-induced neuronal loss by inhibiting inflammation [124]. TMP suppressed inflammatory events possibly by decreasing proinflammatory mediator production and inflammatory cell activation [126]. Due to its antioxidant and anti-inflammatory effects, TMP has been indicated to treat a variety of retinal diseases, particularly DR [127]. In vitro and in vivo investigations indicated that TMP exhibits its effects primarily via protecting neuronal cells from oxidative stress-mediated retinal damage [128,129]. In a recent study, Zhu and colleagues examined the beneficial effect and mechanistic signaling of TMP against IL-1β-induced retinal endothelial cell damage in TR-iBRB2 cells. TR-iBRB2 rat retinal endothelial cells, isolated from the inner BRB, demonstrate the properties characteristic of such a cell barrier [130]. IL-1β mediates dysfunction of TR-iBRB2 cells by stimulating nitrative/oxidative stress; however, this effect was ameliorated by TMP pretreatment of TR-iBRB2 cells. Treatment with TMP protected TR-iBRB2 cells by inhibiting leukostasis, expression of inducible nitric oxide synthase, generation of ROS, mitochondrial dysfunction and mitogen activated protein kinase activation. These results improve our understanding of the protective effect of TMP in DR and will shape the therapeutic development of TMP for its treatment [131].

### 3.16. Sipjeondaebo-Tang

Sipjeondaebo-tang (SDT) is the most extensively used traditional herbal formula to treat anemia, atopic dermatitis, fatigue, loss of appetite, and rheumatoid arthritis in Korea, Japan and China [132]. SDT comprises 12 crude medicinal herbs [133]. SDT possesses several pharmacological activities, including antimicrobial, antioxidant, immunostimulatory and antitumor effects [134]. Yet, the mechanisms by which SDT affects angiogenesis are unclear, and to our knowledge there has been no prior report on retinal pathogenic neovascularization of SDT. Lee and associates explored the effect of SDT on OIR mice, and found that SDT significantly blocked the pathogenic neovascularization and encouraged revascularization of the central retina as compared to the ischemic retinas in the OIR group. SDT dose-dependently and significantly reduced the expression of pro-angiogenic factors (heparin-binding epidermal growth factor-like growth factor, and leptin) in comparison to vehicle-treated OIR mice. Conversely, SDT drastically inhibited anti-angiogenic factors such as thrombospondin-2 and pigment epithelium-derived factor. This implies that SDT elicits its anti-angiogenic effects by suppressing the expression of heparin-binding epidermal growth factor-like growth factor, leptin, and platelet-derived growth factor-AB/BB. Moreover, SDT dose-dependently blocked the interaction between platelet-derived growth factor-BB/platelet- derived growth factor-Rβ [135]. Hence, SDT might be clinically valuable as an herbal medication for ischemic retinopathy. Table 1 indicates pre-clinical efficacy profile of traditional plants in DR.

## 4. Conclusions

Diabetic retinopathy is a major issue and a primary cause of preventable blindness worldwide. Investigations over the past few years have suggested that hyperglycemia drives the onset and development of DR. The mechanisms by which hyperglycemia damages retinal capillaries includes; the amplified polyol pathway, stimulation of protein kinase C, heightened non-enzymatic glycation and production of ROS. An increased understanding of the specific pathological changes in DR will facilitate development of novel therapeutic interventions. TM has established efficacy in, for example, treatment of non-communicable diseases, mental health, disease prevention, and enhancement of the quality of life of persons living with chronic diseases and in the ageing population. Based on the clinical and preclinical data, traditionally used medicinal plants might contain novel oral hypoglycemic compounds, which can counteract the soaring cost and poor availability of existing medicines. However, thorough studies on the safety of plant extracts and their efficacy and mechanisms of action are needed. Pharmaceutical scientists often face problems in developing TM into therapeutics for DR; e.g., toxicity, herb-drug interactions, absence of placebo-controlled randomized clinical trials, product patentability and its standardization. These issues are important concerns for both public and health authorities. National policies regarding TMs are vital for addressing these concerns. The World Health Organization is receiving an increasing number of requests to supply informational and technical guidance to member states regarding national policies on TM. Via regional meetings and publication of documents, World Health Organization is assisting the exchange of information between member states and to facilitate development of national policies concerning TM. Conclusively, considering the long history of herbal TM for the management of DR, our review suggests that natural and traditional herbal medicines have potential as an alternative or combination therapy for DR.

## Figures and Tables

**Table 1:** Evidence-based Traditional Herbal Plant/Formulations in Diabetic Retinopathy.

Plant/Formulation	Active Ingredient/Plant Name	Experimental Evidence	References
*Lactuca sativa*	Phlorizin	Phlorizin noticeably diminishes formation of AGE, retina cell apoptosis and glial fibrillary acidic protein expression in the retinas of *db/db* mice.	[136,137]
*Sparganium stoloniferum*	Sparstolonin B (SsnB)	SsnB blocks the function of endothelial cells associated with angiogenesis in several ex vivo and cellular assays. SsnB restricts endothelial cell division in the G1 phase and negatively regulates the cell cycle proteins cdc6 and cyclin E2. Furthermore, SsnB causes a considerable decrease in blood vessel length and number of branches in the chick chorioallantoic membrane assay.	[138]
*Eriodictyon californicum*	Eriodictyol	Eriodictyol decreases ICAM-1, VEGF, TNF-α, and endothelial nitric oxide synthase in the retina of diabetic rats. Eriodictyol diminishes 40% breakdown of BRB in diabetic rats.	[139]
*Curcuma longa*	Curcumin	Curcumin ameliorates experimental DR in rats via its anti-inflammatory, hypoglycemic, and antioxidant effects.	[140]
*Cyamopsis tetragonoloba*	Genistein	Genistein mitigates retinal inflammation related to diabetes by targeting activation of microglial cells.	[141,142]
*Scutellaria baicalensis*	Baicalein	Baicalein decreases inflammation in a rodent model of DR.	[143]
*Citrus. reticulata*	Hesperidin	Treatment with hesperidin decreases BRB breakdown and augmented retina thickness. Hisperidin decreases blood glucose, aldose reductase and retinal VEGF activity, IL-1β, TNF-α, ICAM-1, and AGEs levels.	[144]
Crude saponin fraction of *P. notoginseng* (CSPN)	Ginsenosides Rg1, Rh1, Rd, and Re	CSPN represses the abnormally increased apoptosis and loss of postsynaptic scaffolding protein PSD-95 by palmitate in staurosporine-differentiated RGC-5 cells. Furthermore, CSPN decreases palmitate-induced endoplasmic reticulum stress-associated eIF2α/ATF4/CHOP, generation of ROS and caspase 12 pathways.	[145]
Green Tea (GT) (*Camellia sinensis*)	Extract of GT	Extract of GT restores retinal anti-oxidant enzymes and decreased proinflammatory factors as compared to a diabetic group.	[48]
*Gaoshan Hongjingtian* (RG)	*Salvia miltiorrhiza*, *Ligusticum chuanxiong Hort* and *Radix Et Rhizoma Glycyrrhizae*	RG inhibits the diabetes-mediated changes in levels of ICAM-1 and NF-κB transcripts. RG attenuates capillary degeneration in diabetic rats. RG diminishes the expression of ICAM-1 and NF-κB in DR. RG inhibits the thickening of basement membrane in retinal capillaries.	[146]
Dang Gui Bu Xue Tang (DBT)	Radix Astragali, Radix *Angelica sinensis* and *Panax notoginseng* (RRP)	RRP suppresses leukostasis, acellular capillaries, and vascular leakage in diabetic rats. RRP reduces the expression of inflammatory factors including MCP-1, ICAM-1, VCAM-1, IL-1β, IL-6, TNF-α, NF-κB, in the retinas of diabetic rats.	[147]
KIOM-79	*Magnolia officinalis*, *Pueraria lobata*, *Glycyrrhiza uralensis* and *Euphorbia pekinensis*	KIOM-79 mitigates AGE-stimulated apoptosis of retinal pericytes by inhibiting NF-κB activation. KIOM-79 exerts its effect via an antioxidant mechanism to ameliorate oxidative stress-induced apoptosis in retinal pericytes.	[148,149]
Danhong Injection (DHI)	Mixture of *Carthamus tinctorius* and *Salvia miltiorrhiza* with main components of tanshinone, tanshinol acid and safflor yellow.	Administration of DHI blocks retinal and retinal sub-layer shrinkage, together with a reduction in AGE. DHI stimulates the expression of fibroblast growth factor 21, peroxisome proliferator-activated γ, and activates the expression of genes responsible for energy expenditure.	[150]
Tangningtongluo formula (TNTL)	Herba Plantaginis, Flos Lonicerae and herba Agrimoniae	Administration of TNTL to *C57BL/KsJ-db/db* mice markedly alleviates the layer thickness of the optic nerve and decreases the density of vascular calibers in fundus oculi.	[151]
He-Ying-Qing-Re Formula (HF)	*Rehmannia glutinosa*, *Lycium barbarum Polygonatum sibiricum*, *Lonicera japonica*, *Angelica sinensis*, *Scrophularia ningpoensis* and *Glycyrrhiza uralensis*.	Experimental studies found HR to inhibit AGE, degeneration of retinal vasculature and BRB permeability damage.	[152]
Buchang NaoXinTong (NXT)	Semen Persicae, *Carthamus tinctorius* L., Frankincense, myrrh, *Spatholobus suberectus*, Achyranthes Root, Cassia Twig, Mulberry Twig, earthworms, scorpions, *Astragalus membranaceus*, *Salvia miltiorrhiza*, *Ligusticum*, Radix Paeoniae Rubra, Szechwan Lovage Rhizome, and *Hirudo*	NXT inhibits the diabetes-induced shrinkage of multiple layers, such as the outer nuclear/plexiform layers and photoreceptor layer in the retina of *db/db* mice.	[153]

## References

[1] Whiting D.R., Guariguata L., Weil C., Shaw J. (2011). IDF diabetes atlas: Global estimates of the prevalence of diabetes for 2011 and 2030. Diabetes Res. Clin. Pract..

[2] Chistiakov D.A. (2011). Diabetic retinopathy: Pathogenic mechanisms and current treatments. Diabetes Metab. Syndr. Clin. Res. Rev..

[3] Scanlon P.H. (2010). Diabetic retinopathy. Medicine.

[4] Shin E.S., Sorenson C.M., Sheibani N. (2014). Diabetes and retinal vascular dysfunction. J. Ophthalmic Vis. Res..

[5] Resnikoff S., Pascolini D., Etya’ale D., Kocur I., Pararajasegaram R., Pokharel G.P., Mariotti S.P. (2004). Global data on visual impairment in the year 2002. Bull. World Health Organ..

[6] Control D., Group C.T.R. (1993). The effect of intensive treatment of diabetes on the development and progression of long-term complications in insulin-dependent diabetes mellitus. N. Engl. J. Med..

[7] Stratton I., Kohner E., Aldington S., Turner R., Holman R., Manley S., Matthews D. (2001). UKPDS 50: Risk factors for incidence and progression of retinopathy in Type II diabetes over 6 years from diagnosis. Diabetologia.

[8] Klein R., Klein B.E., Moss S.E., Cruickshanks K.J. (1994). The Wisconsin epidemiologic study of diabetic retinopathy: XIV. Ten-year incidence and progression of diabetic retinopathy. Arch. Ophthalmol..

[9] Klein R., Knudtson M.D., Lee K.E., Gangnon R., Klein B.E. (2008). The Wisconsin Epidemiologic Study of Diabetic Retinopathy XXII: The twenty-five-year progression of retinopathy in persons with type 1 diabetes. Ophthalmology.

[10] Aiello L.P., Gardner T.W., King G.L., Blankenship G., Cavallerano J.D., Ferris F.L., Klein R. (1998). Diabetic retinopathy. Diabetes Care.

[11] Patel S., Chen H., Tinkham N.H., Zhang K. (2008). Genetic susceptibility of diabetic retinopathy. Curr. Diabetes Rep..

[12] Warpeha K., Chakravarthy U. (2003). Molecular genetics of microvascular disease in diabetic retinopathy. Eye.

[13] Durham J.T., Herman I.M. (2011). Microvascular modifications in diabetic retinopathy. Curr. Diabetes Rep..

[14] Hudson C. (1996). The clinical features and classification of diabetic retinopathy. Ophthalmic Physiol. Opt..

[15] Ola M.S., Nawaz M.I., Siddiquei M.M., Al-Amro S., El-Asrar A.M.A. (2012). Recent advances in understanding the biochemical and molecular mechanism of diabetic retinopathy. J. Diabetes Complicat..

[16] Wilkinson C., Ferris F.L., Klein R.E., Lee P.P., Agardh C.D., Davis M., Dills D., Kampik A., Pararajasegaram R., Verdaguer J.T. (2003). Proposed international clinical diabetic retinopathy and diabetic macular edema disease severity scales. Ophthalmology.

[17] Patel A., Group A.C. (2007). Effects of a fixed combination of perindopril and indapamide on macrovascular and microvascular outcomes in patients with type 2 diabetes mellitus (the ADVANCE trial): A randomised controlled trial. Lancet.

[18] Lang G. (2007). Laser treatment of diabetic retinopathy. Dev. Ophthalmol..

[19] Lewis H. (2001). The role of vitrectomy in the treatment of diabetic macular edema. Am. J. Ophthalmol..

[20] Li D.-D., Chen J.-H., Chen Q., Li G.-W., Chen J., Yue J.-M., Chen M.-L., Wang X.-P., Shen J.-H., Shen X. (2005). Swietenia mahagony extract shows agonistic activity to PPAR gamma and gives ameliorative effects on diabetic *db/db* mice. Acta Pharmacol. Sin..

[21] Al-Rowais N.A. (2002). Herbal medicine in the treatment of diabetes mellitus. Saudi Med. J..

[22] Bailey C.J., Day C. (1989). Traditional plant medicines as treatments for diabetes. Diabetes Care.

[23] Dey L., Attele A. (2002). Alternative therapies for type 2 Diabetes. Altern. Med. Rev..

[24] Head K. (1999). Natural therapies for ocular disorders, part one: Diseases of the retina. Altern. Med. Rev. J. Clin. Ther..

[25] Ceylan-Isik A.F., Fliethman R.M., Wold L.E., Ren J. (2008). Herbal and traditional Chinese medicine for the treatment of cardiovascular complications in diabetes mellitus. Curr. Diabetes Rev..

[26] World Health Organization WHO Traditional Medicine Strategy 2014–2023. http://apps.who.int/iris/handle/10665/92455.

[27] World Health Organization Legal Status of Traditional Medicine and Complementary/Alternative Medicine: A Worldwide Review. http://apps.who.int/medicinedocs/en/d/Jh2943e/.

[28] Patwardhan B., Mashelkar R.A. (2009). Traditional medicine-inspired approaches to drug discovery: Can Ayurveda show the way forward?. Drug Discov. Today.

[29] Hughes B. (2009). 2008 FDA drug approvals. Nat. Rev. Drug Discov..

[30] Kong D.-X., Li X.-J., Zhang H.-Y. (2009). Where is the hope for drug discovery? Let history tell the future. Drug Discov. Today.

[31] Wang Z., Wang J., Chan P. (2013). Treating type 2 diabetes mellitus with traditional Chinese and Indian medicinal herbs. Evid.-Based Complement. Altern. Med..

[32] Kim J., Kim C.-S., Lee Y.M., Sohn E., Jo K., Kim J.S. (2015). Litsea japonica extract inhibits neuronal apoptosis and the accumulation of advanced glycation end products in the diabetic mouse retina. Mol. Med. Rep..

[33] Shin J.Y., Sohn J., Park K.H. (2013). Chlorogenic acid decreases retinal vascular hyperpermeability in diabetic rat model. J. Korean Med. Sci..

[34] Kumar B., Gupta S.K., Nag T.C., Srivastava S., Saxena R., Jha K.A., Srinivasan B.P. (2014). Retinal neuroprotective effects of quercetin in streptozotocin-induced diabetic rats. Exp. Eye Res..

[35] Yu Z., Lu B., Sheng Y., Zhou L., Ji L., Wang Z. (2015). Andrographolide ameliorates diabetic retinopathy by inhibiting retinal angiogenesis and inflammation. Biochim. Biophys. Acta (BBA) Gen. Subj..

[36] Ding Y., Yuan S., Liu X., Mao P., Zhao C., Huang Q., Zhang R., Fang Y., Song Q., Yuan D. (2014). Protective Effects of Astragaloside IV on *db/db* Mice with Diabetic Retinopathy. PLoS ONE.

[37] Kim J.H., Lee Y.M., Ahn E.M., Kim K.W., Yu Y.S. (2009). Decursin inhibits retinal neovascularization via suppression of VEGFR-2 activation. Mol. Vis..

[38] Gong C.-Y., Yu Z.-Y., Lu B., Yang L., Sheng Y.-C., Fan Y.-M., Ji L.-L., Wang Z.-T. (2014). Ethanol extract of Dendrobium chrysotoxum Lindl ameliorates diabetic retinopathy and its mechanism. Vasc. Pharmacol..

[39] Lee S.-Y., Min B.-S., Kim J.-H., Lee J.-K., Kim T.-J., Kim C.-S., Kim Y.-H., Lee H.-K. (2005). Flavonoids from the leaves of Litsea japonica and their anti-complement activity. Phytother. Res..

[40] Min B.S., Lee S.Y., Kim J.H., Kwon O.K., Park B.Y., An R.B., Lee J.K., Moon H.I., Kim T.J., Kim Y.H. (2003). Lactones from the Leaves of *Litsea j aponica* and Their Anti-complement Activity. J. Nat. Prod..

[41] Kim J., Kim C.-S., Lee I.S., Lee Y.M., Sohn E., Jo K., Kim J.H., Kim J.S. (2014). Extract of *Litsea japonica* ameliorates blood-retinal barrier breakdown in *db/db* mice. Endocrine.

[42] Howes M.-J.R., Perry N.S., Houghton P.J. (2003). Plants with traditional uses and activities, relevant to the management of Alzheimer’s disease and other cognitive disorders. Phytother. Res..

[43] Bhatt K., Flora S. (2009). Oral co-administration of α-lipoic acid, quercetin and captopril prevents gallium arsenide toxicity in rats. Environ. Toxicol. Pharmacol..

[44] Bronner C., Landry Y. (1985). Kinetics of the inhibitory effect of flavonoids on histamine secretion from mast cells. Agents Actions.

[45] Fiorani M., de Sanctis R., Menghinello P., Cucchiarini L., Cellini B., Dachà M. (2009). Quercetin prevents glutathione depletion induced by dehydroascorbic acid in rabbit red blood cells. Free Radic. Res..

[46] Chen Y., Li X.-X., Xing N.-Z., Cao X.-G. (2007). Quercetin inhibits choroidal and retinal angiogenesis in vitro. Graefe’s Arch. Clin. Exp. Ophthalmol..

[47] Cao X., Liu M., Tuo J., Shen D., Chan C.-C. (2010). The effects of quercetin in cultured human RPE cells under oxidative stress and in Ccl2/Cx3cr1 double deficient mice. Exp. Eye Res..

[48] Kumar B., Gupta S.K., Nag T.C., Srivastava S., Saxena R. (2012). Green tea prevents hyperglycemia-induced retinal oxidative stress and inflammation in streptozotocin-induced diabetic rats. Ophthalmic Res..

[49] Kumar Gupta S., Kumar B., Srinivasan B., Nag T.C., Srivastava S., Saxena R., Aggarwal A. (2013). Retinoprotective effects of Moringa oleifera via antioxidant, anti-inflammatory, and anti-angiogenic mechanisms in streptozotocin-induced diabetic rats. J. Ocul. Pharmacol. Ther..

[50] Kim J., Kim K.M., Kim C.-S., Sohn E., Lee Y.M., Jo K., Kim J.S. (2012). Puerarin inhibits the retinal pericyte apoptosis induced by advanced glycation end products in vitro and in vivo by inhibiting NADPH oxidase-related oxidative stress. Free Radic. Biol. Med..

[51] Teng Y., Cui H., Yang M., Song H., Zhang Q., Su Y., Zheng J. (2009). Protective effect of puerarin on diabetic retinopathy in rats. Mol. Biol. Rep..

[52] Hsu F.-L., Liu I.-M., Kuo D.-H., Chen W.-C., Su H.-C., Cheng J.-T. (2003). Antihyperglycemic effect of puerarin in streptozotocin-induced diabetic rats. J. Nat. Prod..

[53] Liang X., Xiao S., Lu G., Liang Y., Bi X. (2006). Puerarin protects rat pancreatic islets from damage by hydrogen peroxide. Eur. J. Pharmacol..

[54] Kim J.M., Lee Y.M., Lee G.Y., Jang D.S., Bae K.H., Kim J.S. (2006). Constituents of the roots of *Pueraria lobata* inhibit formation of advanced glycation end products (AGEs). Arch. Pharm. Res..

[55] Liu W., Guo W., Guo L., Gu Y., Cai P., Xie N., Yang X., Shu Y., Wu X., Sun Y. (2014). Andrographolide sulfonate ameliorates experimental colitis in mice by inhibiting Th1/Th17 response. Int. Immunopharmacol..

[56] Hao L.-N., Wang M., Ma J.-L., Yang T. (2012). Puerarin decreases apoptosis of retinal pigment epithelial cells in diabetic rats by reducing peroxynitrite level and iNOS expression. Sheng Li Xue Bao.

[57] Shang X., Pan H., Li M., Miao X., Ding H. (2011). *Lonicera japonica* Thunb.: Ethnopharmacology, phytochemistry and pharmacology of an important traditional Chinese medicine. J. Ethnopharmacol..

[58] Lin L.M., Zhang X.G., Zhu J.J., Gao H.M., Wang Z.M., Wang W.H. (2008). Two new triterpenoid saponins from the flowers and buds of Lonicera japonica. J. Asian Nat. Prod. Res..

[59] Dos Santos M.D., Almeida M.C., Lopes N.P., De Souza G.E.P. (2006). Evaluation of the anti-inflammatory, analgesic and antipyretic activities of the natural polyphenol chlorogenic acid. Biol. Pharm. Bull..

[60] Puupponen Pimiä R., Nohynek L., Meier C., Kähkönen M., Heinonen M., Hopia A., Oksman-Caldentey K.M. (2001). Antimicrobial properties of phenolic compounds from berries. J. Appl. Microbiol..

[61] Shi H., Dong L., Bai Y., Zhao J., Zhang Y., Zhang L. (2009). Chlorogenic acid against carbon tetrachloride-induced liver fibrosis in rats. Eur. J. Pharmacol..

[62] Arion W.J., Canfield W.K., Ramos F.C., Schindler P.W., Burger H.-J., Hemmerle H., Schubert G., Below P., Herling A.W. (1997). Chlorogenic acid and hydroxynitrobenzaldehyde: New inhibitors of hepatic glucose 6-phosphatase. Arch. Biochem. Biophys..

[63] Herling A.W., Burger H.-J., Schubert G., Hemmerle H., Schaefer H.-L., Kramer W. (1999). Alterations of carbohydrate and lipid intermediary metabolism during inhibition of glucose-6-phosphatase in rats. Eur. J. Pharmacol..

[64] De Sotillo D.V.R., Hadley M. (2002). Chlorogenic acid modifies plasma and liver concentrations of: Cholesterol, triacylglycerol, and minerals in (*fa/fa*) Zucker rats. J. Nutr. Biochem..

[65] Lim J.C.W., Chan T.K., Ng D.S.W., Sagineedu S.R., Stanslas J., Wong W.S.F. (2012). Andrographolide and its analogues: Versatile bioactive molecules for combating inflammation and cancer. Clin. Exp. Pharmacol. Physiol..

[66] Li J., Luo L., Wang X., Liao B., Li G. (2009). Inhibition of NF-κB expression and allergen-induced airway inflammation in a mouse allergic asthma model by andrographolide. Cell. Mol. Immunol..

[67] Guan S., Tee W., Ng D., Chan T., Peh H., Ho W., Cheng C., Mak J., Wong W. (2013). Andrographolide protects against cigarette smoke-induced oxidative lung injury via augmentation of Nrf2 activity. Br. J. Pharmacol..

[68] Zhu T., Zhang W., Xiao M., Chen H., Jin H. (2013). Protective role of andrographolide in bleomycin-induced pulmonary fibrosis in mice. Int. J. Mol. Sci..

[69] Zhang W.-J., Hufnagl P., Binder B.R., Wojta J. (2003). Anti-inflammatory activity of astragaloside IV is mediated by inhibition of NF-κB activation and adhesion molecule expression. Thromb. Haemost..

[70] Zheng Z., Liu D., Song C., Cheng C., Hu Z. (1997). Studies on chemical constituents and immunological function activity of hairy root of Astragalus membranaceus. Chin. J. Biotechnol..

[71] Gui D., Guo Y., Wang F., Liu W., Chen J., Chen Y., Huang J., Wang N. (2012). Astragaloside IV, a novel antioxidant, prevents glucose-induced podocyte apoptosis in vitro and in vivo. PLoS ONE.

[72] Liu H., Wei W., Sun W.-Y., Li X. (2009). Protective effects of astragaloside IV on porcine-serum-induced hepatic fibrosis in rats and in vitro effects on hepatic stellate cells. J. Ethnopharmacol..

[73] Lv L., Wu S.-Y., Wang G.-F., Zhang J.-J., Pang J.-X., Liu Z.-Q., Xu W., Wu S.-G., Rao J.-J. (2010). Effect of astragaloside IV on hepatic glucose-regulating enzymes in diabetic mice induced by a high-fat diet and streptozotocin. Phytother. Res..

[74] Motomura K., Fujiwara Y., Kiyota N., Tsurushima K., Takeya M., Nohara T., Nagai R., Ikeda T. (2009). Astragalosides isolated from the root of astragalus radix inhibit the formation of advanced glycation end products. J. Agric. Food Chem..

[75] Yu J., Zhang Y., Sun S., Shen J., Qiu J., Yin X., Yin H., Jiang S. (2006). Inhibitory effects of astragaloside IV on diabetic peripheral neuropathy in rats. Can. J. Physiol. Pharmacol..

[76] Chu Y., Zhang L., Wang X.-Y., Guo J.-H., Guo Z.-X., Ma X.-H. (2011). The effect of Compound Danshen Dripping Pills, a Chinese herb medicine, on the pharmacokinetics and pharmacodynamics of warfarin in rats. J. Ethnopharmacol..

[77] Yang P.J., Li S.M., Lv Y.P., Huang Z.Y., Huang H. (2013). Effect of compound danshen dripping pills on vascular endothelial function in early diabetic retinopathy patients. Chin. J. Exp. Tradit. Med. Formulae.

[78] Lian F., Wu L., Tian J., Jin M., Zhou S., Zhao M., Wei L., Zheng Y., Wang Y., Zhang M. (2015). The effectiveness and safety of a danshen-containing Chinese herbal medicine for diabetic retinopathy: A randomized, double-blind, placebo-controlled multicenter clinical trial. J. Ethnopharmacol..

[79] Ma G., Xu G., Xu L., Wang Z. (1994). Studies on Chemical Constituents of Dendrobium chrysotoxum Lindl. Acta Pharm. Sin..

[80] Ng T.B., Liu J., Wong J.H., Ye X., Sze S.C.W., Tong Y., Zhang K.Y. (2012). Review of research on Dendrobium, a prized folk medicine. Appl. Microbiol. Biotechnol..

[81] Zhao Y., Son Y.-O., Kim S.-S., Jang Y.-S., Lee J.-C. (2007). Antioxidant and anti-hyperglycemic activity of polysaccharide isolated from Dendrobium chrysotoxum Lindl. BMB Rep..

[82] Yu Z., Gong C., Lu B., Yang L., Sheng Y., Ji L., Wang Z. (2015). Dendrobium chrysotoxum Lindl. alleviates diabetic retinopathy by preventing retinal inflammation and tight junction protein decrease. J. Diabetes Res..

[83] Takikawa M., Inoue S., Horio F., Tsuda T. (2010). Dietary anthocyanin-rich bilberry extract ameliorates hyperglycemia and insulin sensitivity via activation of AMP-activated protein kinase in diabetic mice. J. Nutr..

[84] Wu X., Beecher G.R., Holden J.M., Haytowitz D.B., Gebhardt S.E., Prior R.L. (2006). Concentrations of Anthocyanins in Common Foods in the United States and Estimation of Normal Consumption. J. Agric. Food Chem..

[85] Seeram N.P. (2008). Berry fruits: Compositional elements, biochemical activities, and the impact of their intake on human health, performance, and disease. J. Agric. Food Chem..

[86] Nakajima J.-I., Tanaka I., Seo S., Yamazaki M., Saito K. (2004). LC/PDA/ESI-MS profiling and radical scavenging activity of anthocyanins in various berries. BioMed Res. Int..

[87] Colantuoni A., Bertuglia S., Magistretti M., Donato L. (1991). Effects of Vaccinium Myrtillus anthocyanosides on arterial vasomotion. Arzneim.-Forsch..

[88] Lietti A., Cristoni A., Picci M. (1975). Studies on Vaccinium myrtillus anthocyanosides. I. Vasoprotective and antiinflammatory activity. Arzneim.-Forsch..

[89] Matsunaga N., Chikaraishi Y., Shimazawa M., Yokota S., Hara H. (2010). *Vaccinium myrtillus* (Bilberry) extracts reduce angiogenesis in vitro and in vivo. Evid.-Based Complement. Altern. Med..

[90] Kim J., Kim C.-S., Lee Y.M., Sohn E., Jo K., Kim J.S. (2015). *Vaccinium myrtillus* extract prevents or delays the onset of diabetes—Induced blood-retinal barrier breakdown. Int. J. Food Sci. Nutr..

[91] Nalawade S.M., Sagare A.P., Lee C.Y., Kao C.L., Tsay H.S. (2003). Studies on tissue culture of Chinese medicinal plant resources in Taiwan and their sustainable utilization. Bot. Bull. Acad. Sin..

[92] Prakash R.O., Rabinarayan A., Kumar M.S. (2011). *Zingiber zerumbet* (L.) Sm., a reservoir plant for therapeutic uses: A review. Int. J. Res. Ayurveda Pharm..

[93] Yob N., Jofrry S.M., Affandi M., Teh L., Salleh M., Zakaria Z. (2011). *Zingiber zerumbet* (L.) Smith: A review of its ethnomedicinal, chemical, and pharmacological uses. Evid.-Based Complement. Altern. Med..

[94] Hong T.-Y., Tzeng T.-F., Liou S.-S., Liu I.-M. (2016). The ethanol extract of *Zingiber zerumbet* rhizomes mitigates vascular lesions in the diabetic retina. Vasc. Pharmacol..

[95] Raghuram T., Sharma R., Sivakumar B., Sahay B. (1994). Effect of fenugreek seeds on intravenous glucose disposition in non-insulin dependent diabetic patients. Phytother. Res..

[96] Marzouk M., Soliman A., Omar T. (2013). Hypoglycemic and antioxidative effects of fenugreek and termis seeds powder in streptozotocin-diabetic rats. Eur. Rev. Med. Pharmacol. Sci..

[97] Chaturvedi U., Shrivastava A., Bhadauria S., Saxena J.K., Bhatia G. (2013). A mechanism-based pharmacological evaluation of efficacy of trigonella foenum graecum (fenugreek) seeds in regulation of dyslipidemia and oxidative stress in hyperlipidemic rats. J. Cardiovasc. Pharmacol..

[98] Sindhu G., Ratheesh M., Shyni G., Nambisan B., Helen A. (2012). Anti-inflammatory and antioxidative effects of mucilage of Trigonella foenum graecum (Fenugreek) on adjuvant induced arthritic rats. Int. Immunopharmacol..

[99] Middha S., Bhattacharjee B., Saini D., Baliga M., Nagaveni M., Usha T. (2011). Protective role of Trigonella foenum graceum extract against oxidative stress in hyperglycemic rats. Eur. Rev. Med. Pharmacol. Sci..

[100] Kumar P., Kale R., McLean P., Baquer N. (2012). Antidiabetic and neuroprotective effects of Trigonella foenum-graecum seed powder in diabetic rat brain. Prague Med. Rep..

[101] Hur J. (2007). Donguibogam.

[102] Rosenfarb A. (2007). Healing Your Eyes with Chinese Medicine: Acupuncture, Acupressure Chinese Herbs.

[103] Lim J., Kim J., Chung S., Cho S., Oh M., Hwang W. (2009). The Antioxidative and Neuroprotective Effect of Guibi-tang (Guipitang) and Guibi-tang gamibang (Guipitang jiaweijang) on PC12 cells. J. Orient. Neuropsychiatry.

[104] Eun J., Song J. (2004). Effects of Kwibi-tang on serum levels of hormone and the non-specific immune response after immobilization stress in mice. Korean J. Orient. Med. Physiol. Pathol..

[105] Kim H., Choi J., Lim S. (2003). The defensive effect of Keuibi-tang on the gastric mucous membrane of mouse injured by stress and ethanol. J. Orient. Med..

[106] Busta I., Xie H., Kim M.-S. (2009). The use of Gui-Pi-Tang in small animals with immune-mediated blood disorders. Korea Soc. Vet. J..

[107] Lee Y.M., Lee Y.-R., Kim C.-S., Jo K., Sohn E., Kim J.S., Kim J. (2015). Effect of Guibi-Tang, a Traditional Herbal Formula, on Retinal Neovascularization in a Mouse Model of Proliferative Retinopathy. Int. J. Mol. Sci..

[108] So H.S., Oh J., Chung Y.T., Moon Y.J., Kim D.H., Moon B.S., Lee H.S., Baek S.W., Park C., Lim Y.S. (2000). The water extract of Samultang protects the lipopolysaccharide (LPS)/phorbol 12-myristate 13-acetate (PMA)-induced damage and nitric oxide production of C6 glial cells via down-regulation of NF-kappaB. Gen. Pharmacol..

[109] Xie M. (1997). Modern Study of the Medical Formulae in Traditional Chinese Medicine.

[110] Lee Y.M., Kim C.-S., Jo K., Sohn E.J., Kim J.S., Kim J. (2015). Inhibitory effect of Samul-tang on retinal neovascularization in oxygen-induced retinopathy. BMC Complement. Altern. Med..

[111] Yi Z., Dai X., Jiu Y. (1997). Modern Study of Medical Formulae in Traditional Chinese Medicine.

[112] Seo C.-S., Ha H., Jung D.-Y., Lee H.Y., Shin H.-K. (2011). Evaluation of the immune-stimulating activity of Samul-tang, a traditional Korean herbal medicine, standardized by HPLC-PDA. Korean J. Orient. Med..

[113] Kojima S., Inaba K., Kobayashi S., Kimura M. (1996). Inhibitory effects of traditional Chinese medicine Shimotsu-to and its included crude fractions on adjuvant-induced chronic inflammation of mice. Biol. Pharm. Bull..

[114] Yby J.Y.M., Hye Kyung H., Dae Sun H., Hyun Kyoo S. (2008). Subacute toxicity study on Samul-tang in SD rats. Korean J. Orient. Physiol. Pathol..

[115] (2003). State Medical License Number: Z20030017.

[116] Sheng S., Wang Y., Long C., Su W., Rong X. (2014). Chinese medicinal formula Fufang Xueshuantong capsule could inhibit the activity of angiotensin converting enzyme. Biotechnol. Biotechnol. Equip..

[117] Yuan Y.Z., Yuan F., Xu Q.Y., Yu J., Li L., Zhang J.L. (2011). Effect of Fufang Xueshuantong Capsule on a rat model of retinal vein occlusion. Chin. J. Integr. Med..

[118] Cheng Y.X. (2013). Clinical effect observation of compound xueshuantong capsule in the treatment of diabetic retinopathy. GuideChinaMed.

[119] Duan H., Huang J., Li W., Tang M. (2013). Protective effects of fufang xueshuantong on diabetic retinopathy in rats. Evid.-Based Complement. Altern. Med..

[120] Jian W., Yu S., Tang M., Duan H., Huang J. (2015). A Combination of the Main Constituents of Fufang Xueshuantong Capsules Shows Protective Effects against Streptozotocin-induced Retinal Lesions in Rats. J. Ethnopharmacol..

[121] Qian W., Xiong X., Fang Z., Lu H., Wang Z. (2014). Protective effect of tetramethylpyrazine on myocardial ischemia-reperfusion injury. Evid.-Based Complement. Altern. Med..

[122] Tan Z. (2009). Neural protection by naturopathic compounds—An example of tetramethylpyrazine from retina to brain. J. Ocul. Biol. Dis. Inform..

[123] Zhang Z., Wei T., Hou J., Li G., Yu S., Xin W. (2003). Tetramethylpyrazine scavenges superoxide anion and decreases nitric oxide production in human polymorphonuclear leukocytes. Life Sci..

[124] Kao T.-K., Chang C.-Y., Ou Y.-C., Chen W.-Y., Kuan Y.-H., Pan H.-C., Liao S.-L., Li G.-Z., Chen C.-J. (2013). Tetramethylpyrazine reduces cellular inflammatory response following permanent focal cerebral ischemia in rats. Exp. Neurol..

[125] Ren Z., Ma J., Zhang P., Luo A., Zhang S., Kong L., Qian C. (2012). The effect of ligustrazine on l-type calcium current, calcium transient and contractility in rabbit ventricular myocytes. J. Ethnopharmacol..

[126] Liao S.-L., Kao T.-K., Chen W.-Y., Lin Y.-S., Chen S.-Y., Raung S.-L., Wu C.-W., Lu H.-C., Chen C.-J. (2004). Tetramethylpyrazine reduces ischemic brain injury in rats. Neurosci. Lett..

[127] Gong X., Ivanov V.N., Davidson M.M., Hei T.K. (2015). Tetramethylpyrazine (TMP) protects against sodium arsenite-induced nephrotoxicity by suppressing ROS production, mitochondrial dysfunction, pro-inflammatory signaling pathways and programed cell death. Arch. Toxicol..

[128] Liang X., Zhou H., Ding Y., Li J., Yang C., Luo Y., Li S., Sun G., Liao X., Min W. (2012). TMP Prevents Retinal Neovascularization and Imparts Neuroprotection in an Oxygen-Induced Retinopathy ModelTMP Blocks Oxygen-Induced Retinopathy. Investig. Ophthalmol. Vis. Sci..

[129] Ou Y., Dong X., Liu X.-Y., Cheng X.-C., Cheng Y.-N., Yu L.-G., Guo X.-L. (2010). Mechanism of tetramethylpyrazine analogue CXC195 inhibition of hydrogen peroxide-induced apoptosis in human endothelial cells. Biol. Pharm. Bull..

[130] Hosoya K.-I., Tomi M., Ohtsuki S., Takanaga H., Ueda M., Yanai N., Obinata M., Terasaki T. (2001). Conditionally immortalized retinal capillary endothelial cell lines (TR-iBRB) expressing differentiated endothelial cell functions derived from a transgenic rat. Exp. Eye Res..

[131] Zhu X., Wang K., Zhang K., Tan X., Wu Z., Sun S., Zhou F., Zhu L. (2015). Tetramethylpyrazine Protects Retinal Capillary Endothelial Cells (TR-iBRB2) against IL-1β-Induced Nitrative/Oxidative Stress. Int. J. Mol. Sci..

[132] Kogure T., Hoshino A., Ito K., Sato H., Tatsumi T., Ohyama Y., Kawata E., Fujita K.I., Tamura J.I. (2005). Beneficial effect of complementary alternative medicine on lymphedema with rheumatoid arthritis. Mod. Rheumatol..

[133] Shin I.S., Yu Y.B., Seo C.S., Ha H.K., Lee M.Y., Huang D.S., Kim J.H., Shin H.K. (2011). Subchronic toxicity of Sipjeondaebo-tang (SDT) in Sprague-Dawley rats. Regul. Toxicol. Pharmacol..

[134] Tagami K., Niwa K., Lian Z., Gao J., Mori H., Tamaya T. (2004). Preventive effect of Juzen-taiho-to on endometrial carcinogenesis in mice is based on Shimotsu-to constituent. Biol. Pharm. Bull..

[135] Lee Y.M., Kim C.-S., Sohn E., Jo K., Lim H.R., Kim S.K., Kim J.S., Kim J. (2014). Sipjeondaebo-tang, a traditional herbal formula, inhibits retinal neovascularization in a mouse model of oxygen-induced retinopathy. Tohoku J. Exp. Med..

[136] Altunkaya A., Gökmen V. (2009). Effect of various anti-browning agents on phenolic compounds profile of fresh lettuce (L. sativa). Food Chem..

[137] Zhang S.-Y., Li B.-Y., Li X.-L., Cheng M., Cai Q., Yu F., Wang W.-D., Tan M., Yan G., Hu S.-L. (2013). Effects of phlorizin on diabetic retinopathy according to isobaric tags for relative and absolute quantification-based proteomics in *db/db* mice. Mol. Vis..

[138] Bateman H.R., Liang Q., Fan D., Rodriguez V., Lessner S.M. (2013). Sparstolonin B inhibits pro-angiogenic functions and blocks cell cycle progression in endothelial cells. PLoS ONE.

[139] Bucolo C., Leggio G.M., Drago F., Salomone S. (2012). Eriodictyol prevents early retinal and plasma abnormalities in streptozotocin-induced diabetic rats. Biochem. Pharmacol..

[140] Gupta S.K., Kumar B., Nag T.C., Agrawal S.S., Agrawal R., Agrawal P., Saxena R., Srivastava S. (2011). Curcumin prevents experimental diabetic retinopathy in rats through its hypoglycemic, antioxidant, and anti-inflammatory mechanisms. J. Ocul. Pharmacol. Ther..

[141] Subramoniam A. (2016). Plants with Anti-Diabetes Mellitus Properties.

[142] Ibrahim A.S., El-Shishtawy M.M., Peña A., Liou G.I. (2010). Genistein attenuates retinal inflammation associated with diabetes by targeting of microglial activation. Mol. Vis..

[143] Yang L.-P., Sun H.-L., Wu L.-M., Guo X.-J., Dou H.-L., Tso M.O., Zhao L., Li S.-M. (2009). Baicalein reduces inflammatory process in a rodent model of diabetic retinopathy. Investig. Ophthalmol. Vis. Sci..

[144] Shi X., Liao S., Mi H., Guo C., Qi D., Li F., Zhang C., Yang Z. (2012). Hesperidin prevents retinal and plasma abnormalities in streptozotocin-induced diabetic rats. Molecules.

[145] Wang D.-D., Zhu H.-Z., Li S.-W., Yang J.-M., Xiao Y., Kang Q.-R., Li C.-Y., Zhao Y.-S., Zeng Y., Li Y. (2016). Crude Saponins of Panax notoginseng Have Neuroprotective Effects to Inhibit Palmitate-Triggered Endoplasmic Reticulum Stress-Associated Apoptosis and Loss of Postsynaptic Proteins in Staurosporine Differentiated RGC-5 Retinal Ganglion Cells. J. Agric. Food Chem..

[146] Zhao H., Shi X., Wei W., Wang N. (2012). Effect of the regimen of Gaoshan Hongjingtian on the mechanism of poly (ADP-ribose) polymerase regulation of nuclear factor kappa B in the experimental diabetic retinopathy. Chin. Med. J..

[147] Gao D., Guo Y., Li X., Li X., Li Z., Xue M., Ou Z., Liu M., Yang M., Liu S. (2013). An aqueous extract of Radix Astragali, Angelica sinensis, and *Panax notoginseng* is effective in preventing diabetic retinopathy. Evid.-Based Complement. Altern. Med..

[148] Kim J., Kim C.-S., Sohn E., Lee Y.M., Jo K., Kim J.S. (2012). KIOM-79 protects AGE-induced retinal pericyte apoptosis via inhibition of NF-kappaB activation in vitro and in vivo. PLoS ONE.

[149] Kim O.S., Kim J., Kim C.-S., Kim N.H., Kim J.S. (2010). KIOM-79 prevents methyglyoxal-induced retinal pericyte apoptosis in vitro and in vivo. J. Ethnopharmacol..

[150] Liu M., Pan Q., Chen Y., Yang X., Zhao B., Jia L., Zhu Y., Zhang B., Gao X., Li X. (2015). Administration of Danhong Injection to diabetic *db/db* mice inhibits the development of diabetic retinopathy and nephropathy. Sci. Rep..

[151] Cheng L., Meng X.-B., Lu S., Wang T.-T., Liu Y., Sun G.-B., Sun X.-B. (2014). Evaluation of hypoglycemic efficacy of Tangningtongluo formula, a traditional Chinese Miao medicine, in two rodent animal models. J. Diabetes Res..

[152] Wang L., Wang N., Tan H.-Y., Zhang Y., Feng Y. (2015). Protective effect of a Chinese Medicine formula He-Ying-Qing-Re Formula on diabetic retinopathy. J. Ethnopharmacol..

[153] Zhang F., Huang B., Zhao Y., Tang S., Xu H., Wang L., Liang R., Yang H. (2013). BNC protects H9c2 cardiomyoblasts from H_2_O_2_-induced oxidative injury through ERK1/2 signaling pathway. Evid.-Based Complement. Altern. Med..

